# LLM-Conductor: A Closed-Loop Resource-Adaptive Architecture for Secure LLM Deployment in Industrial Sensor Networks and IIoT Systems

**DOI:** 10.3390/s26092733

**Published:** 2026-04-28

**Authors:** Kai Xu, Diming Zhang, Xuguo Wang

**Affiliations:** 1 School of Computer Science and Engineering, Jiangsu University of Science and Technology, Zhenjiang 212100, China; 232211901235@stu.just.edu.cn; 2 State Key Laboratory for Novel Software Technology, Nanjing University, Nanjing 210046, China; xuguo.wang@nju.edu.cn

**Keywords:** LLM security, industrial deployment, closed-loop control, resource optimization, industrial environment simulation and adaptation

## Abstract

To address the bottlenecks of missing decision-making closed loop, insufficient experience reuse, and decoupled resource scheduling in industrial LLM deployment, this paper proposes LLM-Conductor, a three-layer collaborative architecture that enables monitoring-feedback autonomous decision-making, structured policy memory, and joint policy-resource optimization.Through ablation studies, horizontal comparisons with ISOLATEGPT and ReAct, and graded resource-reduction experiments across six tiers, the results demonstrate that the security risk incidence rate is reduced from 70.6 percent to 1.3 percent, the multi-application collaborative task completion rate reaches 100 percent, and token utilization improves to 88.9 percent. Under constraints of at least 512 MB memory and at least 0.5 GHz CPU, the core task completion rate remains above 95 percent. By deeply coupling decision-making with resource scheduling, this architecture provides an integrated pathway toward efficient, secure, and reliable LLM deployment in Industrial Internet of Things scenarios. Current validation focuses on software-layer interaction patterns under simulated resource-constrained environments, with physical-layer industrial integration reserved for future work.

## 1. Introduction

In recent years, large language models (LLMs) have evolved rapidly from standalone text-generation tools into agentic systems capable of multi-task collaboration, third-party tool integration, and cross-scenario interaction [1,2,3]. Their automation paradigm, driven by natural language instructions, has demonstrated significant value across various real-world scenarios [4]. For instance, users can employ natural language to instruct an LLM to invoke toolchains for automatically executing complex planning [5] or natural language processing tasks [6]. While this end-to-end intelligent collaboration is propelling LLMs from research prototypes toward industrial deployment, it simultaneously brings core challenges—such as deployment efficiency, resource adaptability, and system security—to the forefront of practical implementation.

However, the widespread industrial deployment of LLM applications still faces significant bottlenecks, which can be deconstructed into three critical issues. First, the absence of a decision-making closed loop results in insufficient adaptive capability. Traditional LLMs are prone to catastrophic forgetting and policy failures in dynamic environments [7,8]. Second, there is a lack of experience reuse mechanisms and existing toolchain optimization is often one-sided. This leads to repeated exploration in similar tasks, increasing response latency, and the selection of toolchains tends to ignore global resource constraints, resulting in high task interruption rates in resource-constrained environments [5,9,10]. Third, a prominent contradiction exists between deployment invasiveness and system observability. Current monitoring solutions either require code instrumentation, incurring significant performance overhead, or lack sufficient data fusion capability to support effective fault diagnosis [11,12]. Existing approaches primarily focus on optimizing a single dimension (e.g., resource scheduling [13] or memory mechanisms [14]), or they fail to account for the unique complexities of LLM deployment scenarios, such as multi-tool collaboration and environmental dynamics. Consequently, they fail to provide a systematic solution, which severely hinders the reliable industrial deployment of LLMs.

To address the challenges outlined above, this paper proposes the LLM-Conductor architecture. Its core concept is a three-layer collaborative framework comprising Policy Optimization, Foundational Support, and Theoretical Guarantee. This design aims to tackle the following three central challenges: (1) establishing a monitoring-feedback decision-making closed loop under the constraint of zero-intrusive deployment; (2) enabling cross-task experience reuse and global optimization of toolchains; and (3) ensuring adaptable task execution in resource-constrained environments. The ultimate goal is to achieve the research vision of efficient autonomy, resource adaptability, and reliable deployment.

The paper conducts ablation studies using an unoptimized Vanilla-LLM-App baseline to validate the marginal contributions of each core module across the following three dimensions: security defense, functional integrity, and resource efficiency. It also performs horizontal comparisons against ISOLATEGPT [12], the standard ReAct Agent [15], and the CostBench [16] industry benchmark to highlight the differentiated advantages of the active monitoring-feedback closed loop, structured policy memory, and dynamic joint optimization. Additionally, graded resource-reduction experiments are constructed via VMware with six descending configuration tiers, simulating extreme environments from edge nodes to micro-sensors to determine the architecture’s operational boundaries and adaptation capabilities. The entire evaluation framework is based on the LangChain Benchmarks, real-world scenario cases, and a 2004-case security test suite, completing quantitative assessment through three rounds of repeated experiments under strictly controlled software and hardware environments.

Ablation experiments demonstrate that, compared with the unoptimized baseline system, LLM-Conductor reduces the security risk incidence rate from 70.6% to 1.3%, decreases the output format error rate from 28.6% to 4.8%, effectively eliminates cross-application data mismatch, and improves token utilization from approximately 33% to above 85%. Comparative experiments further reveal that the architecture outperforms ISOLATEGPT in security (risk incidence rate: 1.3% vs. 8.5%; defense response time: 78.9 ms vs. over 2000 ms), significantly surpasses the standard ReAct Agent in functional integrity (multi-application collaborative task completion rate improved from approximately 65% to 100%; step accuracy improved from between 0.42 and 0.62 to 1.0; cross-application data mismatch reduced from between 16% and 22% to 0%), and exceeds the CostBench fixed-quota policy in resource efficiency (token utilization increased from approximately 55% to 88.9%), collectively demonstrating its empirical benefits for efficient, secure, and reliable LLM deployment in industrial scenarios.

In single-task adaptation tests across six graded resource tiers, task completion rates remained at 100% for Tiers 1–3, 95% for Tier 4, and 74% for the extreme Tier 5, with failures occurring only in Tier 6. Response latency, along with peak CPU and memory utilization, increased gradually across tiers before stabilizing, indicating the framework’s adaptation stability in constrained scenarios. These tests establish the operational boundary at **1 CPU core, 1 thread, 512 MB RAM, and a 0.5 GHz clock frequency**.

The core contributions of this paper are summarized as follows:

**C1:** A cross-layer coupling mechanism for closed-loop monitoring-feedback-decision. It integrates zero-intrusive collection (psutil/pynvml/watchdog), anomaly detection (Isolation Forest/DBSCAN), and LLM-driven policy generation into an end-to-end “perception-analysis-decision-execution-feedback” loop, enabling direct triggering of dynamic LLM policy adjustments by monitoring anomalies. It further defines empirical criteria for zero-intrusive observability via feature completeness, overhead constraint, and zero-intersection constraint, elevating non-intrusive deployment from engineering practice to an empirically verifiable framework.

**C2:** A structured policy memory and cross-task transfer mechanism. Unlike conventional vector databases storing raw dialogue text, it leverages RedisGraph to construct a policy-level heterogeneous graph (task-tool-parameter-result), enables cross-task policy reuse via TF-IDF scenario matching, and designs a success-rate-threshold-driven incremental writing mechanism, overcoming the lack of policy-level structured organization in existing memory systems.

**C3:** Joint policy-resource optimization under resource constraints. Existing approaches typically decouple tool selection from resource scheduling. This work employs the Qwen tokenizer for precise counting, MDP/PPO for global optimization, and a three-level elastic degradation for resource adaptation. By explicitly embedding resource consumption into the MDP state space and reward function, it achieves global joint optimization of toolchain composition and resource allocation, establishing a quantitative policy-resource mapping to maintain core task completion rates ≥ 95% under resource constraints.

**C4:** Quantified adaptation boundaries for resource-constrained environments. Through six-level gradient experiments from edge nodes to micro-sensors, it quantitatively demonstrates that core functionalities maintain 95% completion rates under ≥512 MB memory and ≥0.5 GHz CPU, and 100% rates under ≥2 GB memory and ≥1.0 GHz CPU. This clarifies operable boundaries for industrial edge deployment and provides quantitative evidence for reliable LLM deployment in IoT scenarios.

## 2. Motivation and Related Work

### 2.1. Research Motivation

Current LLM applications face industry pain points across the following four primary dimensions:

**Lack of Decision-Making Closed Loop Leading to Weak Adaptive Capability.** When making autonomous decisions in dynamic environments, LLMs lack an effective closed-loop mechanism, resulting in insufficient adaptive capability. Traditionally, knowledge acquisition by LLMs for specific tasks relies on distinct training phases, which molds them into static knowledge entities that struggle to effectively update or adjust their strategies when tasks change [7,17]. For instance, while LLMs possess strong reasoning abilities, deploying them in real-world autonomous driving scenarios faces challenges such as high cost and latency [18]. Concurrently, traditional machine learning methods demonstrate poor adaptability in autonomous decision-making tasks [8,19].

**Scarcity of Experience Reuse Mechanisms Constraining Decision Efficiency.** LLM applications often repeat the policy exploration process for similar tasks, failing to effectively reuse historical decision-making experience. For example, in a medical-travel coordination service, handling a returning patient’s follow-up appointment and flight booking requires re-invoking the toolchain, leading to fragmented cross-session data [5]. When solving specific decision-making tasks, the absence of a task-specific experience reuse mechanism limits decision efficiency, making effective adaptation difficult under limited task data [20].

**Toolchain Optimization Lacking a Global Perspective.** Existing methods commonly employ a greedy policy to select tool invocation sequences, overlooking resource constraints and efficiency balance across the entire task lifecycle. Particularly in resource-constrained environments, unreasonable tool invocation can significantly increase the probability of resource exhaustion while substantially reducing task completion rates [9,10,21].

**Prominent Contradiction in Resource Adaptability.** Due to their large model size and high computational complexity, LLMs face significant challenges when deployed on resource-constrained devices. Fixed resource allocation struggles to respond to real-time resource fluctuations [22].

In response to these limitations, we introduce the LLM-Conductor framework. Its goal is to address the shortcomings of current approaches in achieving closed-loop decision-making and co-optimizing resource allocation with policy execution.

### 2.2. Related Work

The following section reviews the limitations of related work and presents our solutions, organized from five perspectives.

#### 2.2.1. LLM Autonomous Decision-Making Framework

- **Rule-driven architectures** (e.g., LangChain Agent [23], ADRD [24]) suffer from limitations such as reliance on predefined templates and decision failures in low-frequency scenarios. LLM-Conductor addresses these by establishing a monitoring-feedback-driven autonomous decision-making architecture. This creates an end-to-end closed loop that integrates machine learning-based anomaly detection with LLM deep reasoning, enabling the real-time generation of adaptive policies.
- **Reinforcement learning-driven approaches** (e.g., AutoGPT [25], MORAL [26]) are often plagued by slow convergence and high resource consumption. LLM-Conductor mitigates these issues by constructing a policy memory graph to enable cross-task experience reuse, thereby reducing inefficient exploration. It employs the PPO algorithm for offline pre-training followed by online fine-tuning, balancing global optimization with convergence efficiency.
- **Hybrid decision architectures** (e.g., MetaGPT [27]) typically lack a feedback closed loop and struggle to adapt to real-time resource fluctuations. LLM-Conductor introduces a closed-loop mechanism that spans the entire decision-making process, allowing for real-time perception of resource and task states to dynamically adjust toolchain combinations and parameters.

#### 2.2.2. Memory Mechanisms and Experience Reuse

- **In-session Memory.** This type is primarily reflected within the context window. For instance, ChatGPT (GPT-4)’s ability to maintain context within a single conversation [28] allows it to directly leverage the model’s internal attention mechanisms to process current session information [29]. Its main limitations are the constraint of context window length and the inability to reuse experiences across sessions. LLM-Conductor overcomes these by implementing a cross-session memory persistence mechanism. It uses Redis to categorically store dialogue history, entity information, and policy graphs, thereby breaking the context window constraint and enabling cross-session experience reuse.
- **External Memory Systems.** These systems typically store relevant information, such as historical interaction data, in external storage like vector databases [14]. However, they still face significant challenges in the structured organization of data, the design of cross-task transfer mechanisms, and the efficiency of matching similar tasks. LLM-Conductor addresses these through its policy memory graph. Leveraging a Redis-based structure that incorporates task types and scenario features, it achieves efficient cross-task transfer.

#### 2.2.3. Multi-Tool Coordination and Resource Optimization

- **Tool Selection Strategies.** Existing strategies often focus on single-step decisions, failing to effectively balance global task optimization objectives with resource constraints. For example, frameworks like OTC [30] exhibit a local optimization-oriented design that can lead to sub-optimal behaviors. Similarly, frameworks like ReAct [31], which rely on incremental decision processes, are prone to falling into local optima. In contrast, our architecture models the toolchain invocation sequence as a Markov Decision Process (MDP) and employs PPO reinforcement learning to achieve global optimization, thereby avoiding the pitfalls of single-step decision-making and local optima.
- **Resource Scheduling Methods.** Significant progress has been made in resource allocation for fog computing and IoT environments, as well as in resource management strategies for mobile edge computing [13]. However, in complex multi-step tool usage scenarios, existing resource allocation methods often fail to adequately consider the overall efficiency and cost-effectiveness of the entire toolchain. For instance, the CostBench benchmark [16] suffers from insufficient integration between resource scheduling and toolchain policy. Our architecture addresses this by implementing dynamic resource allocation based on precise token counting and a tiered-threshold elastic scheduling mechanism, enabling real-time awareness of resource load and the pursuit of optimal overall toolchain utility.

#### 2.2.4. Zero-Intrusion Deployment and Observability

**Zero-intrusive solutions** (e.g., CloudProcMon [11]) and zero-trust deployment theories (e.g., ISOLATEGPT [12]) struggle to support end-to-end root-cause analysis for faults and lack a formalized definition of observability. In contrast, LLM-Conductor addresses this by constructing a multi-source data fusion engine that enables comprehensive data correlation across the entire workflow. This approach not only facilitates precise root-cause localization but also provides a clear implementation pathway for achieving observability under a zero-intrusion paradigm.

#### 2.2.5. From Prompt Heuristics to Structured Coupling

Recent studies indicate that in complex dynamic environments, such as IoT and edge infrastructure, LLM coordination mechanisms relying solely on prompts are vulnerable to context drift and semantic ambiguity, resulting in reasoning fragility. To mitigate this issue, IoT-LLM [32] employs IoT-oriented retrieval-augmented generation and chain-of-thought prompting to explicitly associate raw sensor data with structured domain knowledge, rather than merely relying on prompts to activate commonsense; Sigfrid [33] leverages LLM causal reasoning combined with structured rule representation (the Tapen prompting methodology) to perform formalized interference detection among rules before deployment, likewise transcending pure prompt heuristics.

In contrast, LLM-Conductor adopts a hybrid paradigm that couples prompt-driven generation with runtime structured constraints as follows: the policy memory graph dynamically constructs heterogeneous experience networks via RedisGraph to enable structured knowledge reuse; MDP joint optimization explicitly embeds resource constraints within a 32-dimensional state space, imposing structured boundaries on LLM decisions through PPO; and the monitoring-feedback closed loop encodes raw observations as structured summaries fed into the model. Consequently, LLM-Conductor anchors LLM reasoning within system resource boundaries through runtime memory topology and state-space constraints, exhibiting stronger robustness and reproducibility than pure prompt-based strategies.

### 2.3. Summary of Limitations

Based on the above analysis, existing research on LLM application optimization exhibits three notable shortcomings as follows:

(1) **At the decision-making level,** existing frameworks mostly rely on rule-based templates or incremental single-step reasoning, lacking a closed-loop mechanism for runtime monitoring and dynamic feedback, making it difficult to achieve adaptive policy adjustment amid environmental fluctuations.
(2) **At the memory level,** existing solutions are confined to vector storage of raw dialogue text or single-session context windows, failing to achieve structured organization of policy-level experience and efficient cross-task transfer.
(3) **At the resource level,** tool selection policy is decoupled from resource scheduling, and existing methods mostly adopt local greedy or fixed-quota modes, unable to achieve global joint optimization of toolchain composition and resource allocation under resource-constrained conditions.

LLM-Conductor addresses these limitations by integrating the aforementioned technical aspects through its three-layer architecture. This integration forms an end-to-end solution that comprehensively covers decision-making, memory, and resource management, thereby providing a novel technical pathway for the efficient deployment of LLMs in complex scenarios.

## 3. Architecture

**Figure 1:**
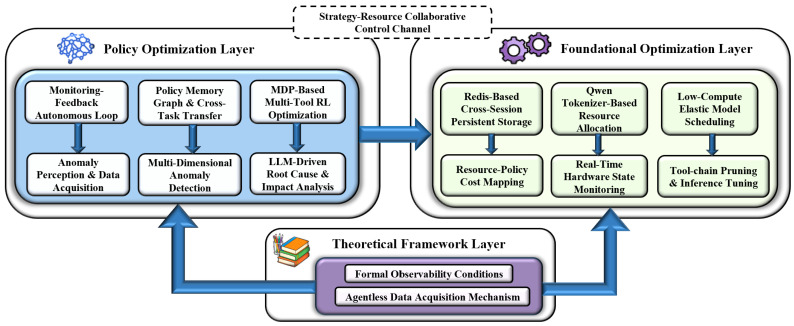
Three-layer collaborative optimization framework for LLM-driven task scheduling: the Policy Optimization Layer handles decision-level anomaly processing and cross-task transfer; the Bottom Optimization Layer executes resource-oriented scheduling and hardware monitoring; and the Theoretical Framework Layer underpins observability and data acquisition. The “policy-Resource Collaborative Control Channel” enables inter-layer bidirectional interaction.

As shown in Figure 1, LLM-Conductor is a monitoring-feedback-driven autonomous decision-making framework designed for LLM applications. It focuses on addressing the challenges of efficiency, reliability, and resource adaptation in deploying LLMs within complex environments. Its core employs a **three-layer collaborative architecture—Policy Optimization, Foundational Support, and Theoretical Guarantee**—which orchestrates the entire workflow through a **“Perception-Analysis-Decision-Action-Feedback” closed-loop logic**, delivering an end-to-end solution. Specifically, the Policy Optimization Layer serves as the decision-making core, generating adaptive policies based on monitoring data. The Foundational Support Layer provides resource adaptation and data persistence capabilities, ensuring stable operation in resource-constrained environments. The Theoretical Guarantee system defines the boundaries of observability and deployment specifications through zero-intrusion deployment theory. These three layers interact via shared data and control buses, forming a dynamically iterative closed-loop operational mechanism.

In terms of engineering implementation, the framework is developed in Python. Its core engine is built upon LangChain and vLLM, utilizes Redis for cross-session memory persistence, and supports both command-line interaction and API invocation. Centered on the core logic of **“Three-layer Collaboration + Closed-loop Drive + Resource Self-adaptation”**, the framework achieves a dual enhancement of decision efficiency and system security while maintaining compatibility with resource-constrained environments.

### 3.1. Design of the Policy Optimization Layer

The policy optimization layer serves as the core innovation carrier of this framework, achieving intelligent policy generation, experience reuse, and efficient optimization through three key technologies. These correspond to contributions C1 and C2 proposed in the introduction.

#### 3.1.1. Monitoring-Feedback Decision Loop

The monitoring-feedback decision loop overcomes the limitations of traditional static decision-making through the following four stages: monitoring data collection, multi-dimensional analysis, adaptive decision-making, and execution feedback. Designed under the zero-intrusion principle, this mechanism integrates machine learning anomaly detection with large language model reasoning to achieve comprehensive system state perception, dynamically generate adaptive strategies, and iteratively optimize through feedback.

Zero-intrusion data collection gathers system states at a sampling frequency of 1 Hz via read-only kernel interfaces, producing structured monitoring records. Log tracking operates through filesystem event monitoring without placing write locks on original files.

The anomaly detection module employs the following two complementary unsupervised algorithms:

- **Isolation Forest** is employed for resource anomaly detection, configured with n_estimators = 100 and contamination = 0.05 (anomaly ratio). The model is trained on initial baseline data, with inference latency below 1 ms per sample.
- **DBSCAN** is utilized for log anomaly detection, vectorizing cleaned log text via TfidfVectorizer with parameters eps = 0.5, min_samples = 5, and metric = “cosine”. Samples not belonging to any cluster are classified as anomalies.

The system interfaces with the Qwen large language model (deployed via vLLM) for anomaly interpretation, constructing structured prompts to convert ML anomaly results into natural language analysis. The policy generator maps anomaly types to executable operations, supporting dynamic threshold adjustment (e.g., CPU threshold default 80%, GPU threshold default 95%), thereby forming a complete closed loop of “collection-detection-analysis-decision-execution-feedback”.

#### 3.1.2. Policy Memory Graph and Cross-Task Transfer Mechanism

The policy memory graph and cross-task transfer mechanism utilize a graph database as the underlying infrastructure, constructing a structured graph containing task types, scenario features, tool invocation sequences, and execution outcomes. This achieves cross-session memory persistence and historical policy reuse through scenario feature matching.

The graph is organized into four node types—Task, Tool, Parameter, and Outcome—connected by usage and dependency edges (USES, DEPENDS_ON, LEADS_TO). It is constructed through offline initialization from historical data followed by online runtime updates.

Cross-task transfer matches scenario descriptions via TF-IDF vectorization and cosine similarity. Policy reuse is triggered when similarity exceeds 0.7, directly retrieving the historical optimal tool invocation chain. Transfer latency is constrained within 50 ms through batch querying and local caching. The graph update policy employs incremental writing: strategies are persisted only when the task success rate exceeds 90% and execution time is below average, thereby avoiding noise data pollution.

#### 3.1.3. MDP-Based Multi-Tool Policy Reinforcement Learning Optimization

The Markov Decision Process (MDP)-based multi-tool policy reinforcement learning optimization models tool invocation sequences as sequential decision problems, achieving global optimization through reinforcement learning.

##### MDP Formulation

The state space S represents the joint space of resource states (CPU, memory, and GPU utilization), task states (task type, current step, and historical tool list), and constraint states (remaining budget and timeout threshold), with a state dimensionality of 32. The action space A comprises 20 fundamental tool combinations (e.g., search engine, code interpreter, and knowledge graph query) and parameter variants (temperature coefficient 0.0–1.0 and sampling quantity 1–5), resulting in a discrete action space of size 100.

The reward function R(s,a) balances execution efficiency, resource consumption, and task success rate as follows:

$$R\left(s,a\right)=r_{time}+r_{res}+r_{success}$$

where $r_{time}=-0.1\times t_{exec}$ represents the execution time penalty, $r_{res}=-0.01\times \left(cpu+mem\right)$ represents the resource consumption penalty, and $r_{success}=10$ (if successful) or −5 (otherwise) represents the task outcome reward.

##### Algorithm Implementation

The system adopts the Proximal Policy Optimization (PPO) algorithm. Policy optimization proceeds in two stages as follows: an offline pre-training phase in a simulated environment covering diverse task categories and resource load levels, followed by online fine-tuning every 10 completed real tasks to dynamically adjust tool invocation order and parameters, thereby maximizing cumulative rewards.

### 3.2. Design of the Foundational Support Layer

The foundation support layer provides core runtime infrastructure for the framework, addressing resource constraints in resource-constrained environments through three key technologies. These correspond to contributions C2, C3, and C4 proposed in the introduction.

**Cross-Session Memory Persistence Technology.** Cross-session memory persistence technology partitions memory data into the following three categories: complete conversation history, entity information, and core summaries, stored in dedicated database namespaces. Through hybrid RDB + AOF persistence, connection pooling, and pipeline batch operations, seamless cross-session data reuse is achieved. Memory read latency is stably controlled within 100 ms through local caching and serialization optimization.

**Context-Aware Dynamic Resource Allocation Technology.** Context-aware dynamic resource allocation technology employs a model-specific tokenizer for accurate token counting, with empirical error below 3%, dynamically allocating output quotas based on model context length and redundancy thresholds to avoid overflow errors.

Dynamic allocation follows a three-step logic as follows: (1) calculate input text token count $N_{input}$; (2) evaluate task complexity (simple/medium/complex) to determine base reservation; (3) compute available output quota $N_{output}=\min\left(N_{model\_max}-N_{input}-N_{safety},N_{task\_limit}\right)$, where $N_{model\_max}=4096$ (Qwen context limit), $N_{safety}=500$ (safety threshold), and $N_{task\_limit}$ is dynamically set by task type (simple: 512; medium: 2048; complex: 8192). This quota is dynamically injected at runtime without initialization hardcoding, ensuring strict alignment with the vLLM service API specifications.

**Elastic Model Scheduling for Resource-Constrained Environments.** Elastic model scheduling for resource-constrained environments adopts joint policy-resource modeling, continuously monitoring computing status and dynamically adjusting scheduling strategies. A resource consumption prediction model quantifies expected execution cost from current system load and task type, triggering three-level elastic degradation:

- **Level 1** (Sufficient resources): Enables full-function toolchain (20 tools including code interpreter and complex knowledge graph queries);
- **Level 2** (Resource-constrained): Switches to simplified toolchain (10 core tools, disabling heavy computation modules);
- **Level 3** (Resource-critical): Retains only lightweight tool combination (three basic tools: search engine, text summarization, simple calculation).

The system supports task priority scheduling (real-time tasks P0 can preempt resources from ordinary tasks P1, with preemption latency <50 ms). Under resource-constrained conditions, this solution maintains task completion rate above 95% through dynamic degradation and load balancing, improving resource utilization by over 30% compared to static resource allocation strategies.

### 3.3. Theoretical Guarantee System

The preceding layers rely on comprehensive runtime state perception to drive decision-making and resource scheduling. However, conventional monitoring solutions often require code instrumentation or intrusive system calls, which compromise the stability of host LLM services. To address this, we present an empirical zero-intrusion observability framework (corresponding to contribution C1). This framework establishes an engineering practice with explicit overhead thresholds, hierarchical isolation boundaries, and reproducible verification methods. It is important to clarify that this section does not provide formal mathematical proofs of system correctness; rather, it defines practical constraints and empirical criteria that ensure monitoring activities do not perturb the host application, validated through runtime measurements and system-level traces.

#### 3.3.1. Zero-Intrusion Collection Protocol

The framework achieves non-intrusive data acquisition through three hierarchical isolation mechanisms, implemented in the LLM-Conductor monitoring module.

**System Call Layer Isolation.** The collector invokes kernel read-only interfaces via psutil and nvidia-ml-py3, accessing /proc/stat, /proc/{pid}/stats, and NVML driver interfaces. These operations are strictly read-only, with a default sampling frequency of 1 Hz, single acquisition latency below 5 ms, CPU utilization under 0.3%, and memory footprint below 50 MB.

**Filesystem Layer Isolation.** Based on watchdog, the framework implements inotify event monitoring. It tracks file offsets (seek() to EOF) to read only appended log content, adopting a copy-on-read policy to ensure no write locks are placed on original log files. Log processing throughput exceeds 10 MB/s with latency below 100 ms.

**Process Space Isolation.** The monitoring process runs in a separate execution context from the host LLM service (vLLM deployment). It obtains port and process information via standard library queries (psutil.net_connections and psutil.process_iter), avoiding ptrace system calls. The collection operation set is restricted to read, open, and fork operations, with no write, inject, or attach operations.

#### 3.3.2. Observability Data Design and Sampling Constraints

To ensure that the collected data sufficiently represents runtime states without formal proofs of completeness, we establish engineering constraints on feature coverage and sampling rates.

The monitoring feature vector is constructed as follows:

$$m_{t}=\left[CPU_{usage},\;MEM_{usage},\;GPU_{0,usage},\;GPU_{1,usage},\;Port_{conn},\;L_{t-k:t},\;I_{anomaly}\right]$$

where the log window $L_{t-k:t}$ retains raw logs from the most recent k=30 s (30 sampling points). Guided by the Nyquist sampling theorem ($f_{s}\geq 2f_{max}$, where $f_{max}=0.5$ Hz is the typical anomaly frequency of LLM services), the 1 Hz sampling rate provides alias-free state reconstruction for resource anomalies within the observed bandwidth. This is a **design constraint** rather than a formal guarantee of state-space coverage.

#### 3.3.3. Performance Isolation and Adaptive Overhead Control

To enforce explicit overhead boundaries, the framework employs resource hard limits and adaptive sampling strategies.

Resource hard limits are enforced via cgroups or OS-level quotas as follows: the monitoring process is constrained to a CPU quota of at most 10%, a memory limit of 512 MB, and disk I/O priority set to idle (ionice -c 3). When host service resources are constrained (CPU usage >90%), the framework automatically down-samples to 0.2 Hz (every 5 s). This adaptive policy ensures that the expected processing cost remains below the predefined overhead threshold $\Theta_{overhead}$ with empirical probability p>0.95 under tested workloads.

The overhead threshold $\Theta_{overhead}$ defaults to 5% of host CPU time and 1% of GPU memory bandwidth. These thresholds are **empirically determined** based on the resource sensitivity of LLM inference workloads and represent operational constraints rather than formally verified bounds.

#### 3.3.4. Empirical Verification Methods

Since the framework provides engineering-level isolation rather than formal proofs, we define reproducible verification procedures to validate its non-intrusive properties.

**Zero-Intrusion Verification.** Executing strace -e trace = write,ptrace -p $(pgrep llm_conductor) returns empty results, indicating the absence of intrusive system calls at the syscall trace level.

**Overhead Threshold Verification.** The monitoring process CPU utilization and memory footprint are measured under baseline and stressed host conditions using standard system utilities to verify compliance with $\Theta_{overhead}$.

**Data Consistency Verification.** The md5sum hash of original log files is computed before and after the monitoring period. Unchanged checksums confirm append-only writes and the absence of file modification by the monitoring process.

### 3.4. Prompt Construction and System Observation Encoding

To ensure reproducibility of the LLM-driven decision-making process, this subsection details the prompt construction pipeline and the encoding of system observations before they are fed into the Qwen model.

**Dynamic Template Nature.** The prompt employed in the monitoring-feedback loop is **dynamically constructed at runtime** rather than relying on a static template. It is assembled through a three-stage encoding pipeline that converts heterogeneous raw observations into structured natural language.

**Stage 1: Raw Structured Collection.** The DataCollector module gathers system states at a sampling frequency of $f_{s}=1$ Hz, producing structured JSON records covering system resources, GPU status, process states, network connections, and log streams.

**Stage 2: Statistical Aggregation.** The aggregation routine extracts the most recent 60 records (60 s sliding window) and computes averaged resource metrics, process status summaries, and anomaly detection readiness flags.

**Stage 3: Natural Language Encoding.** The aggregated results are encoded into a structured natural language prompt comprising four sections: a role definition, a monitoring summary of system and GPU resources, ML anomaly detection results, and actionable task instructions. The exact prompt template is omitted here for brevity.

**Fault-Tolerant Prompt Design.** If certain monitoring dimensions are unavailable due to hardware absence or insufficient baseline data, the prompt automatically switches to degraded natural-language descriptions, preserving structural integrity and preventing parser failures.

### 3.5. Token-Aware Input Encoding and Dynamic Context Allocation

This subsection defines empirical criteria for the token-level encoding of the planner input and the dynamic allocation of output token budgets.

**Composition of the Full Input Text *P*.** Before tokenization, the complete prompt *P* fed to the planner is concatenated from four ordered segments as follows:

1. **Cross-task reuse rules:** A hard-coded instruction block (approximately 300 tokens) enforcing entity extraction and parameter filling constraints.
2. **Dialogue history:** Retrieved from short-term memory and formatted as alternating user-assistant lines.
3. **Current user query:** The raw natural-language request.
4. **Tool descriptions:** Structured text containing tool names, functional descriptions, and parameter schemas.

**Tokenizer and Token Counting.** We employ the Qwen model tokenizer, which implements Byte-Pair Encoding (BPE) optimized for Chinese corpora. The input length is obtained by encoding the complete prompt, with empirical verification against actual vLLM inference consumption indicating a token-counting error margin below 3%.

**Dynamic Quota Calculation.** Given the model context limit $N_{model\_max}=4096$ and a safety reserve $N_{safety}=500$, the available output quota is computed as follows:

$$N_{available}=N_{model\_max}-N_{input}-N_{safety}$$

$$N_{output}=\max\left(\min\left(N_{available},1000\right),\;500\right)$$

The upper bound (1000) prevents overallocation under extreme input lengths, while the lower bound (500) ensures a minimally viable response. This value is dynamically injected at runtime without hard-coded initialization, ensuring alignment with the inference service API specifications.

**Task-Adaptive Limits.** Additionally, $N_{output}$ is further capped by task-type limits $N_{task\_limit}$ (simple: 512; medium: 2048; complex: 8192), ensuring that the final allocation satisfies:

$$N_{final}=\min\left(N_{output},N_{task\_limit}\right)$$

## 4. Implementation

To comprehensively validate the overall performance of the LLM-Conductor framework in terms of effectiveness, security, functional completeness, and resource adaptability, this section integrates the methodologies of controlled experiments, case studies, and quantitative analysis to design and conduct multi-dimensional experiments. Guided by the contribution objectives C1–C3, the core advantages of LLM-Conductor are systematically evaluated through ablation experiments with baseline system comparisons, real-world scenario case validation, and standardized benchmark testing.

All experimental results are derived from actual runtime data, including vLLM service logs, case execution records, system monitoring metrics, and resource utilization trends, thereby ensuring the credibility and reproducibility of the conclusions. This experimental design fully satisfies the requirements of academic research for rigor, systematicity, and transparency.

### 4.1. Experimental Objectives

This experiment focuses on the following four core objectives:

(1) To verify the security of the monitoring-feedback-driven autonomous decision-making architecture and the zero-intrusion deployment theory, specifically the framework’s defense capability against malicious attacks and unintentional data leakage.
(2) To validate the functional completeness of the policy memory graph and MDP-based multi-tool optimization, i.e., the framework’s ability to complete tasks in both single-application and multi-application collaboration scenarios without functional degradation.
(3) To quantify the performance and resource efficiency of dynamic resource allocation and elastic model scheduling, in terms of response latency, throughput, and resource utilization.
(4) To verify the adaptability of the framework in resource-constrained environments, specifically its ability to maintain stable operation and task completion rates under limited computational resources.

### 4.2. Experimental Environment Configuration

To ensure the reproducibility of the experiments, the software and hardware environment along with dependency configurations are detailed as follows:

- **Hardware Environment:** The hardware configuration consists of an Intel Xeon Gold 5317 CPU (32 cores, 3.00 GHz), 128 GB of RAM, two NVIDIA A40 GPUs (each with 48 GB VRAM), and a 2 TB SSD. The system supports AVX512 instructions and NUMA architecture, and it runs on a virtualized platform with CUDA 13.0 and corresponding NVIDIA drivers.
- **Software Environment:** The operating system is Ubuntu 22.04 LTS with kernel version 5.15.0-78-generic. Core dependencies include Python 3.9.16, LangChain 0.1.10, vLLM 0.4.0, Redis 7.0, scikit-learn 1.3.2, Stable Baselines3 2.0.0, psutil 5.9.8, and pynvml 11.5.0. The large language model Qwen2-72B-Instruct is deployed using the vLLM service with tensor parallelism (across 2 GPUs). The service is configured with a GPU memory utilization of 0.95 and a maximum context length of 4096, along with specific monitoring addresses, port numbers, and API access keys.
- **Additional Software Dependencies for Reproducibility:** The prompt encoding and memory modules rely on the transformers library (version 4.39.0) for tokenizer loading. The cross-session memory stack explicitly employs the following three LangChain memory classes:

ConversationBufferMemory for short-term full-context retention, ConversationSummaryBufferMemory for long-term summarization, and a custom subclass QwenConversationSummaryBufferMemory that overrides token-counting methods to use the Qwen tokenizer. The dynamic allocation constants are fixed as MAX_CONTEXT_LENGTH=4096 and RESERVE_TOKENS=500.

### 4.3. Baseline System and Datasets

To ensure experimental rigor, we provide a detailed description of the comparison systems and datasets.

**A. Control System and Ablation Variants:**

To establish a rigorous experimental control benchmark, we constructed Vanilla-LLM-App as the control system. This system maintains full functional equivalence to LLM-Conductor while excluding all optimization layers, emulating the default deployment mode of conventional LLM applications. Specifically, it removes the policy optimization layer (including the monitoring-feedback loop, policy memory graph, and MDP-based reinforcement learning) and the infrastructure optimization layer (including elastic scheduling and dynamic token allocation), replacing them with a fixed resource allocation policy and shared memory storage.

Furthermore, to independently evaluate the actual contributions of the proposed encoding and coupling mechanisms, we constructed four ablation variants with explicit removal of the following components: (1) dynamic prompt construction is replaced by a fixed static template without machine learning anomaly injection or structured observation encoding; (2) the statistical aggregation pipeline is bypassed so that raw JSON snapshots are passed directly without 60-s sliding-window summarization; (3) token-aware input encoding is disabled by fixing max_tokens = 1000 regardless of input length, eliminating tokenizer-based adaptive allocation; and (4) monitoring-decision coupling is removed by decoupling system monitoring from the LLM planner to serve only as passive logging. These controlled removals ensure that any performance differences between the control system and the complete architecture are strictly attributable to the encoding and coupling mechanisms themselves, rather than underlying model capacity.

**B. Experimental Datasets:**

**(1) Real-World Scenario Case Set**

This set comprises four representative tasks that simulate core interaction patterns in LLM-based industrial applications. As summarized in Table 1, these scenarios are structurally isomorphic to typical IIoT deployment workflows at the software-interaction layer. The set covers single-application usage, multi-application collaboration, and cross-session data reuse scenarios.

**Representative input/output examples:** For **Travel Expense Calculation**, a sample query is as follows: “*Calculate the fare from Main Street to Elm Avenue using both metro and ride-sharing options.*” The expected output is a structured comparison containing estimated fares, distances, and durations from both tools.

For **Email Summarization**, a sample query is as follows: “*Summarize the latest email from my inbox.*” The system retrieves real email data via the QQ Email POP3 interface and returns the subject, sender, timestamp, and cleaned body summary.

For **Medical-Tourism**, the workflow for these three rounds of dialogue is as follows: in the first round, symptoms and available appointment slots are asked for; in the second round, the time of the appointment is confirmed; and in the third round, all the information is compiled into a structured list.

For **Symptom Consultation**, a sample query is as follows: “*I am experiencing fatigue and persistent pain.*” The expected output includes symptom analysis (e.g., “likely chronic fatigue syndrome”), medical recommendations, and precautions.

**Representativeness of real-world scenarios:** While these scenarios originate from consumer-grade applications, they are structurally isomorphic to typical IIoT deployment patterns as follows: (i) single-tool deterministic queries mirror industrial parameter retrieval (e.g., querying a sensor’s current reading); (ii) unstructured data ingestion emulates industrial log and alert stream processing; (iii) cross-domain multi-agent coordination is structurally identical to workflows where a Manufacturing Execution System interacts with supply-chain and quality-control databases; and (iv) knowledge-based consultation mirrors fault diagnosis in predictive maintenance. We acknowledge that these scenarios do not involve physical PLCs, OT protocols, or Modbus telemetry; however, they capture the software-layer interaction patterns—toolchain invocation, cross-session data reuse, resource contention under concurrent requests, and cross-application data isolation—that are generalizable across IIoT LLM deployments. Domain-specific validation involving SCADA logs and industrial fieldbus protocols is reserved for future work.

**(2) Standardized Benchmark Dataset**

Constructed based on LangChain Benchmarks [34], this dataset comprises four types of query tasks: 42 queries without tools (NAQ), 20 single-application queries (SAQ), 10 multi-application queries (MAQ), and 21 multi-application collaboration queries (MACQ). It is used to supplement functional validation and resource scheduling experiments. These benchmarks are peer-reviewed and widely adopted in LLM-agent evaluations; we employ them to ensure comparability with the existing literature.

**(3) Security Test Set**

This set comprises 2004 cases from the following three sources: 1054 base cases and 544 extended cases from the ISOLATEGPT-Sec security assessment dataset [12] and InjecAgent [35], and 406 independently extended cases targeting multi-application collaboration risks. From a risk typology perspective, the set is classified into the following seven categories: Malicious Toolchain Tampering (MTT), Sensitive Data Exfiltration (SDE), Unintentional Data Leakage (UDL), Functional Anomalies caused by Natural Language Ambiguity (FA-NLA), Resource Exhaustion Attacks (REA), Prompt Injection Attacks (IIA), and Cross-Application Privilege Abuse (CPA). The latter two categories (IIA and CPA) extend ISOLATEGPT-Sec’s original five-class framework to cover risks specific to multi-agent collaborative environments.

**Construction of the 406 independently extended cases.** These 406 cases are generated through a hybrid policy to ensure coverage of attack vectors unique to industrial IoT as follows:

*(i) Manual crafting (200 cases).* Following the MITRE ATLAS framework for LLM adversarial tactics, two authors manually designed industrial-specific threats, including toolchain tampering with PLC parameters, sensor data poisoning through prompt injection, and cross-device privilege escalation in multi-agent environments.

*(ii) Automated generation via mutation engine (206 cases).* A script-based engine applies **12 attack templates** (prompt injection, privilege escalation, resource exhaustion, cross-application data exfiltration, toolchain hijacking, natural language ambiguity exploitation, etc.) to the four real-world scenario tasks in this paper (transportation cost calculation, email summarization, medical-travel collaboration, symptom consultation), generating 3–5 semantic variants per template. All automatically generated cases were independently verified by two authors before inclusion to ensure syntactic correctness, attack feasibility, and non-duplication with existing benchmarks.

## 5. Experiments

This section evaluates LLM-Conductor across three dimensions—security, functional integrity, and resource efficiency—through two complementary experimental perspectives. Ablation studies against the unoptimized Vanilla-LLM-App baseline isolate the marginal contributions of each core module. Horizontal comparisons against ISOLATEGPT, the standard ReAct Agent, and CostBench position the architecture against external benchmarks under controlled variables.

All experiments draw from the LangChain Benchmarks, real-world scenario cases, and a 2004-case security test suite, executed under unified hardware and software configurations with multiple repetitions to ensure reproducibility.

### 5.1. Ablation Experiments

To rigorously validate the individual contributions and synergistic effects of LLM-Conductor’s core modules, this section conducts ablation studies using the unoptimized Vanilla-LLM-App as the control system. Four ablation variants are constructed, each disabling one specific mechanism (dynamic prompt construction, statistical aggregation, token-aware encoding, and monitoring-decision coupling). These experiments isolate internal performance differences and attribute them to the proposed encoding and coupling mechanisms, rather than to underlying model capabilities. The evaluation covers the following three dimensions: security defense (C1), functional integrity (C2), and resource efficiency (C3).

#### 5.1.1. Security Ablation Experiment

This experiment evaluates the defensive efficacy of the monitoring-feedback loop and zero-intrusive deployment by comparing the complete architecture against the control system (Vanilla-LLM-App, with the policy optimization layer fully removed) and Ablation Variant 4 (monitoring decoupled from decision-making, reduced to passive logging). Using the 2004-case security test suite, it quantifies risk occurrence rate, anomaly detection rate, defense response time, and false positive rate to attribute security performance differences to the monitoring-decision coupling mechanism.

**Experimental Setup.** The three systems are deployed under identical hardware and model configurations. All 2004 test cases are executed via automated batch submission with real-time state monitoring and timeout handling for resource exhaustion attacks.

**Evaluation Metrics.** Risk occurrence rate (proportion of triggered risks among all cases), average data leakage per case (number of illegally acquired sensitive items), anomaly detection rate (proportion of identified anomalies), defense response time (duration from detection to countermeasure execution, in ms), and false positive rate (proportion of benign requests incorrectly flagged). The latter three metrics apply only to LLM-Conductor and its ablation variant, as the baseline lacks monitoring and defense modules.

**Figure 2:**
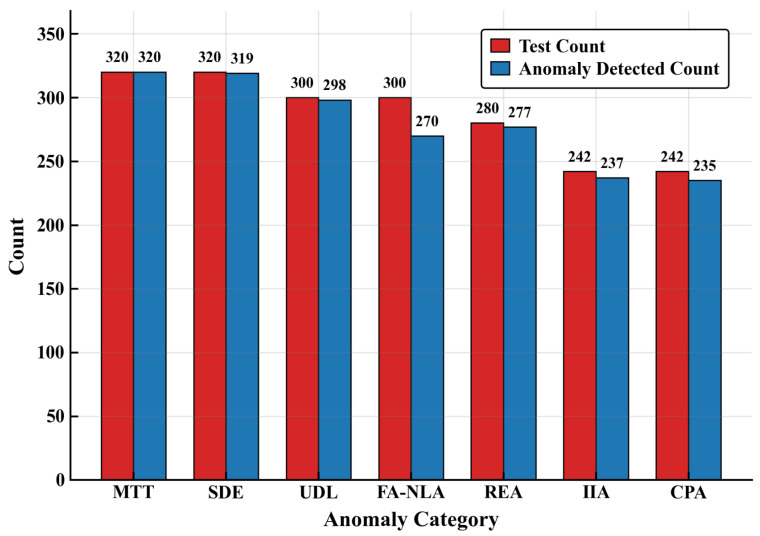
LLM-Conductor statistical histogram of the number of tests and the number of anomaly detections for different risk types.

**Findings.** The results of this experiment are presented in Table 2 and Table 3, and Figure 2, covering overall security indicators, per-category risk analysis, and anomaly detection counts.

As shown in Table 2, LLM-Conductor reduces the average risk occurrence rate from 70.6% to 1.3%, and it suppresses average data leakage from 3.2 to 0.02 items per case, with no successful attacks observed. Its anomaly detection rate reaches 96.7% (surpassing the 90% threshold), defense response time is 78.9 ms (below the 100 ms real-time threshold), and false positive rate is 1.8% (better than the 5% requirement), all satisfying practical operational benchmarks.

Table 3 details protective performance across seven risk categories. Occurrence rates remain minimal for explicit attacks such as Malicious Toolchain Tampering (0.0%) and Sensitive Data Exfiltration (0.2%), and stay low even for covert risks including Function Anomaly due to Natural Language Ambiguity (2.3%) and Instruction Injection Attack (2.7%). The narrow 95% Wilson confidence intervals (e.g., 0.0–1.2% for MTT) indicate statistically stable protection. Figure 2 further confirms high detection consistency, with detected anomaly counts closely matching test case counts across categories (e.g., 320 of 320 for MTT; 319 of 320 for SDE). These results indicate that the monitoring-decision coupling mechanism provides substantial and empirically measurable defensive benefits.

In summary, the security ablation results empirically support the effectiveness of the monitoring-feedback closed loop and zero-intrusive deployment.

#### 5.1.2. Functional Integrity Ablation Experiment

This experiment evaluates the contribution of structured policy memory and cross-task transfer mechanisms to functional integrity. It compares the complete architecture against the control system (with the policy memory graph and MDP-based reinforcement learning removed) and two ablation variants as follows: Variant 1 (fixed static template replacing dynamic prompt construction) and Variant 2 (bypassing statistical aggregation). Task completion capabilities are assessed in single-application and multi-application collaboration scenarios, attributing performance differences to the policy memory and context encoding mechanisms.

**Experimental Setup.** Six task categories from the LangChain framework [34] and custom scenarios are employed as follows:

(1) **SA-TCC:** Query transportation fares via *metro_hail* and *quick_ride*.
(2) **SA-ES:** Extract and summarize the latest email.
(3) **MAC-MT:** Schedule a medical appointment and coordinate flight booking.
(4) **SA-SC:** Provide advisory suggestions for “fatigue and persistent pain”.
(5) **LC-AI:** Structured information extraction from 42 email texts.
(6) **LC-MAC:** Cross-tool data association on 21 relational entries.

All cases are executed on both Vanilla-LLM-App and LLM-Conductor with uniformly configured inputs, recording per-step operational logs and final outputs.

**Evaluation Metrics. Intermediate Step Accuracy** (proportion of correct tool invocations per step), **Task Completion Rate** (proportion of cases producing expected outputs), and **Error Type Distribution** (proportion of each error category among failed cases).

**Findings.** As shown in Table 4, both systems perform identically on SA-TCC. LLM-Conductor achieves Intermediate Step Accuracy and Task Completion Rate of 1.0 on SA-ES, SA-SC, and MAC-MT, significantly improving upon the Vanilla system’s low performance in multi-application collaboration. For LC-MAC, step accuracy is enhanced while completion remains comparable to the baseline. LC-AI shows similar performance in both systems, indicating that optimizations do not compromise basic functionality.

Table 5 details the error distribution. LLM-Conductor reduces “Output Format Errors” from 28.6% to 4.8% via the Policy Memory Graph, and it eliminates “Cross-application Data Mismatch” entirely through the MDR Context Isolation Module. Errors stemming from inherent LLM limitations (e.g., context length overflow) remain comparable across both systems. These results indicate that structured policy memory and context encoding substantially improve functional integrity in application-invocation tasks.

These findings indicate that structured policy memory and MDP-based optimization substantially enhance functional integrity in multi-application tasks.

#### 5.1.3. Performance and Resource Efficiency Ablation Experiment

This experiment evaluates the contribution of resource co-optimization and elastic scheduling mechanisms to resource efficiency and operational stability. It compares the complete architecture against the control system (with the infrastructure optimization layer removed and replaced by fixed resource allocation) and Ablation Variant 3 (token-aware input encoding disabled, max_tokens fixed at 1000), attributing performance differences to the proposed encoding and scheduling mechanisms.

**Experimental Setup.** Four task types from the LangChain framework are employed as follows: Application-Independent Queries, Multi-Application Collaborative Queries, Multi-Application Queries, and Single-Application Queries. For each task type, all test cases are executed on both systems, synchronously recording response time, actual token usage, maximum allocated tokens, and hardware utilization (CPU, memory, GPU).

**Figure 3:**
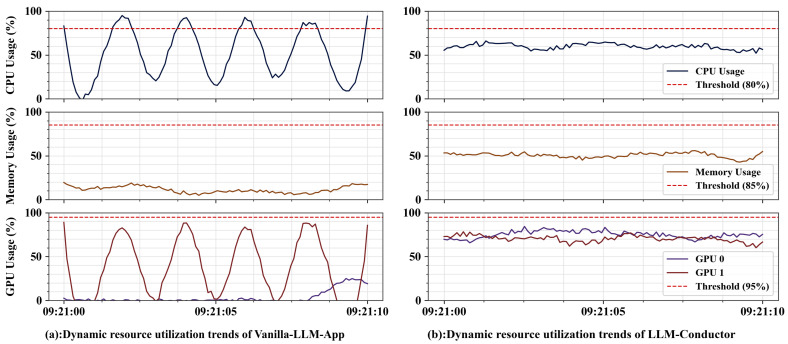
Comparative analysis of dynamic trends in CPU, memory, and GPU resource utilization under experimental conditions for the two architectures.

**Findings.** The results are shown in Table 6 and Figure 3. As shown in Table 6, LLM-Conductor achieves token resource utilization exceeding 85% across all task scenarios, reaching 92.4% ± 2.5% in multi-application collaborative queries. In contrast, Vanilla-LLM-App exhibits decreased utilization as task complexity increases, dropping to 25.9% ± 5.1% in the same scenario. The average response time of LLM-Conductor is slightly higher than the baseline, reflecting the reasonable overhead of precise resource scheduling. The substantial improvement in token utilization—averaging over 50—signifies a shift from fixed quotas to adaptive, on-demand resource provisioning.

Figure 3 further reveals the hardware load patterns. Vanilla-LLM-App exhibits sharp, spiky CPU fluctuations and severe GPU allocation imbalance (GPU 1 frequently approaching 100% while GPU 0 remains below 20%). In contrast, LLM-Conductor stabilizes CPU utilization at approximately 60% (consistently below the 80% threshold) and synchronously balances dual-GPU loads within the 60–80% range, maintaining full-dimensional resource utilization below threshold limits. These results indicate that dynamic resource allocation and elastic scheduling substantially improve resource efficiency and operational stability.

Overall, dynamic resource allocation and elastic scheduling demonstrate measurable gains in token utilization and hardware stability.

#### 5.1.4. Ablation Synthesis

The ablation experiments collectively indicate that the proposed mechanisms independently contribute to their respective target dimensions: monitoring-decision coupling improves security defense, structured policy memory enhances functional integrity, and dynamic joint optimization increases resource efficiency. These internal attributions are further positioned against external benchmarks in Section 5.2.

### 5.2. Comparative Experiments

While Section 5.1 isolates internal contributions via controlled removal of modules, this section conducts horizontal comparisons against three external baselines—ISOLATEGPT (security), the standard ReAct Agent (functionality), and CostBench (resource efficiency)—to demonstrate the competitive advantages of the full architecture. All comparisons strictly control hardware, model versions, and datasets to ensure that observed differences reflect architectural paradigm distinctions (active closed-loop vs. passive isolation; global planning vs. incremental decision-making; dynamic joint optimization vs. fixed-quota allocation).

#### 5.2.1. Security Comparison

**Experimental Objective.** This experiment contrasts LLM-Conductor’s active monitoring-feedback closed loop against ISOLATEGPT’s passive isolation paradigm under controlled variables, comparing risk incidence, response speed, deployment invasiveness, and performance overhead.

**Experimental Setup.** Both systems are deployed on identical hardware and software configurations; only the security mechanism differs. The 2004-case security test set (1054 ISOLATEGPT-Sec base cases, 544 InjecAgent extensions, and 406 cross-application privilege abuse cases) is executed on both systems. ISOLATEGPT is configured per its NDSS 2025 release with process isolation and privilege mediation; LLM-Conductor deploys the full three-layer architecture with zero-intrusive monitoring.

**Findings.** As shown in Table 7, ISOLATEGPT exhibits a risk incidence rate of 8.5%, significantly higher than LLM-Conductor’s 1.3%. This gap stems from ISOLATEGPT’s inability to perceive implicit attacks (e.g., semantic ambiguity, resource exhaustion) that do not trigger privilege dialogs, whereas LLM-Conductor’s closed-loop monitoring detects such behaviors via DBSCAN log analysis and Isolation Forest resource profiling. Defense response time is compressed from over 2000 ms (manual confirmation) to 78.9 ms (automated closed-loop), with an anomaly detection rate of 96.7% and a false positive rate of 1.8%. ISOLATEGPT requires host-level system call modifications, resulting in high invasiveness and a 92.4% latency increase in multi-application collaboration due to IPC overhead and cold-start. LLM-Conductor operates through read-only interfaces with performance penalty below 3%.

**Figure 4:**
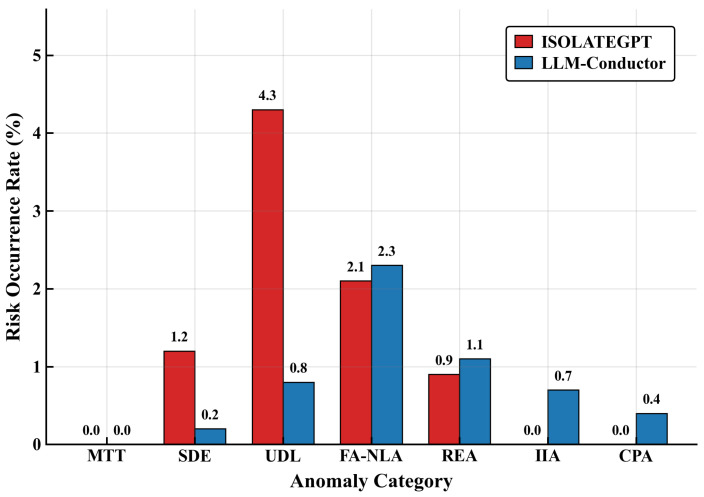
Per-category risk incidence comparison between ISOLATEGPT and LLM-Conductor.

As shown in Figure 4, ISOLATEGPT achieves absolute blocking (0%) against explicit cross-application attacks (MTT, IIA, and CPA), yet it remains unaware of implicit attacks (UDL 4.3%, SDE 1.2%). LLM-Conductor suppresses these to 0.8% and 0.2%, respectively. Both systems struggle against semantic ambiguity (FA-NLA 2.1% vs. 2.3%), and resource exhaustion rates are comparable (0.9% vs. 1.1%).

***Note:*** The per-category risk incidence in Table 7 deviates slightly from the ablation results in Table 2, as the two experiments employ distinct control-variable strategies. The ablation study exposes the full attack surface on a unified native runtime, whereas the horizontal comparison co-locates both systems where ISOLATEGPT’s micro-isolation substrate pre-filters explicit attack vectors at the system-call level, altering the residual distribution reaching the application-layer detector. Both settings consistently demonstrate that LLM-Conductor achieves substantial risk suppression.

Overall, LLM-Conductor’s aggregate risk incidence of 1.3% is superior to ISOLATEGPT’s 8.5%, primarily because implicit attacks dominate industrial scenarios and closed-loop monitoring possesses perception capability against such “invisible attacks” that isolation architectures inherently fail to detect.

#### 5.2.2. Functional Comparison

**Experimental Objective.** This experiment validates the functional advantages of LLM-Conductor’s structured policy memory and cross-task transfer mechanism over standard ReAct incremental single-step decision-making in multi-application collaborative tasks. The LangChain built-in ZeroShotAgent (ReAct mode) [15,31] serves as the baseline, utilizing the identical Qwen2-72B-Instruct backend and the same toolset.

**Experimental Setup.** The ReAct Agent is deployed on the same vLLM service with a consistent toolset and the same dataset as in ablation experiment 5.1.2, recording tool invocation sequences and final outputs.

**Evaluation Metrics.** *Task Completion Rate* (proportion of final outputs conforming to expectations); *Intermediate Step Accuracy* (proportion of correct tool invocation sequences and parameter fillings); *Cross-Application Data Mismatch Rate (CADM)* (proportion of data field errors or losses during cross-tool transmission); and *Output Format Error Rate (OFE)* (proportion of final outputs not conforming to the expected Schema).

**Findings.** As shown in Table 8, LLM-Conductor maintains task completion rate and intermediate step accuracy above 0.96 for all six tasks, with four achieving perfect scores. In contrast, ReAct drops sharply to 0.42–0.62 and 0.52–0.78 in multi-application collaborative tasks (MAC-MT and LC-MAC), indicating that incremental single-step decision-making suffers from severe error accumulation during cross-tool orchestration. Regarding CADM, LLM-Conductor achieves 0% across all tasks, whereas ReAct reaches 16–22% in multi-application tasks, demonstrating that structured memory eliminates field errors and data loss through explicit data dependency modeling. For OFE, LLM-Conductor is nearly 0%, while ReAct fluctuates between 6–18%, reflecting that global policy planning ensures output Schema compliance whereas local single-step reasoning struggles to maintain final format consistency.

This performance gap stems from ReAct’s reliance on local optimal matching at each step, where minor deviations in early steps are amplified exponentially in subsequent steps when multi-tool data association and long-range dependencies are involved. LLM-Conductor, conversely, pre-constructs a global execution blueprint through the policy optimization layer, encoding tool invocation sequences, data mapping relationships, and output format requirements into structured memory, enabling all steps to operate collaboratively under unified policy constraints. These results indicate that global policy planning replacing incremental single-step decision-making provides a scalable reliability assurance mechanism for LLM-based agent systems in complex tasks.

#### 5.2.3. Resource Efficiency Comparison

**Experimental Objective.** This experiment positions LLM-Conductor’s dynamic joint optimization against fixed-quota strategies, using CostBench [16] as the external baseline and Vanilla-LLM-App as the same-environment control.

**Experimental Setup.** Data are sourced from the ablation experiment in Section 5.1.3, with external benchmark data added under identical conditions.

**Findings.** As shown in Table 9, CostBench reports average token utilization of approximately 55% under fixed-quota strategies. Vanilla-LLM-App achieves only 32.9% in the same environment, dropping to 25.9% in MACQ complex collaborative tasks. Through context-aware tokenizer counting and task-complexity grading, LLM-Conductor raises average utilization to 88.9%—an absolute improvement of 33.9 percentage points over CostBench and 56.0 percentage points over Vanilla-LLM-App. In MACQ scenarios, utilization reaches 92.4%, improving by 44.4 percentage points over CostBench and 66.5 percentage points over Vanilla-LLM-App.

These results indicate that fixed-quota strategies fail to resolve resource waste in complex multi-turn tasks. LLM-Conductor’s dynamic joint optimization elevates token utilization from ∼55% to 88.9%, demonstrating a paradigm shift from “functionally available” to “resource-efficient” deployment.

### 5.3. Cross-Dimensional Experimental Synthesis

The ablation experiments isolate the contributions of individual internal modules, while the comparative experiments position these mechanisms against external baselines. Together, they demonstrate that deep coupling of policy and resource layers constitutes a key paradigm shift for LLM industrial deployment.

- **Security:** Compared with ISOLATEGPT’s passive isolation, LLM-Conductor’s monitoring-feedback closed loop suppresses implicit attack risks (UDL/SDE) from 4.3%/1.2% to 0.8%/0.2%, upgrading defense from boundary isolation to behavioral perception.
- **Functionality:** Against standard ReAct’s incremental single-step decision-making (step accuracy dropping to 0.42–0.62 in multi-app tasks), structured policy memory maintains accuracy above 0.96 and eliminates cross-application data mismatch.
- **Efficiency:** Against fixed-quota baselines (CostBench ∼55%, Vanilla-LLM-App 32.9%), dynamic joint optimization raises average token utilization to 88.9%.

In summary, the marginal contribution of LLM-Conductor lies in the empirical benefits of policy-resource coupling: closed-loop monitoring enables implicit threat detection, structured memory ensures controllability in complex tasks, and dynamic joint optimisation delivers efficiency under resource constraints. The synergy of these three elements forms a comprehensive technical pathway for autonomous decision-making, resource adaptation, and reliable deployment.

## 6. Resource-Constrained Adaptation Experiment

### 6.1. Introduction to the Experiment

To validate the deployment capability of the LLM-Conductor architecture in resource-constrained environments typical of edge computing and Industrial Internet of Things (IIoT) devices [36,37,38], this section employs a methodology combining graded resource reduction tests, comparative ablation, and parameter sensitivity analysis. By incrementally downgrading configuration parameters to simulate resource-constrained environments, we conduct a quantitative analysis of the architecture’s core functionalities. This process assesses its functional integrity and resource scheduling efficiency, thereby predicting its operational performance in real-world low-resource scenarios and deducing its adaptation boundaries.

This experiment is conducted on VMware [39], employing a gradually decreasing hardware gradient design. CPU resources (cores, threads, frequency) and memory are selected as the core variables, while configurations for GPU and storage are controlled synchronously to uphold the principle of controlling variables.

Six gradient levels of resource-constrained environments are established, as detailed in Table 10. These levels span from edge computing nodes to minimal IoT sensors, covering typical low-resource scenarios and ensuring the scientific validity and representativeness of the experimental design. The gradient design methodology is as follows: Gradients 1–4 are used to demonstrate adaptation capability, while Gradients 4–6 employ an ablation-comparison approach to identify the adaptation boundaries under resource constraints.

**Virtualization Configuration Specification**: Hardware resource reduction across gradients was achieved through VMware by limiting CPU frequency, disabling CPU cores, partitioning and isolating video memory, and allocating fixed memory quotas. All environments were configured with a consistent software stack: Ubuntu 22.04 LTS, Python 3.9.16, LangChain 0.1.10, vLLM 0.4.0, and Redis 7.0. This setup ensures that computational resources are the sole experimental variable.

### 6.2. Task Case Design

**Figure 5:**
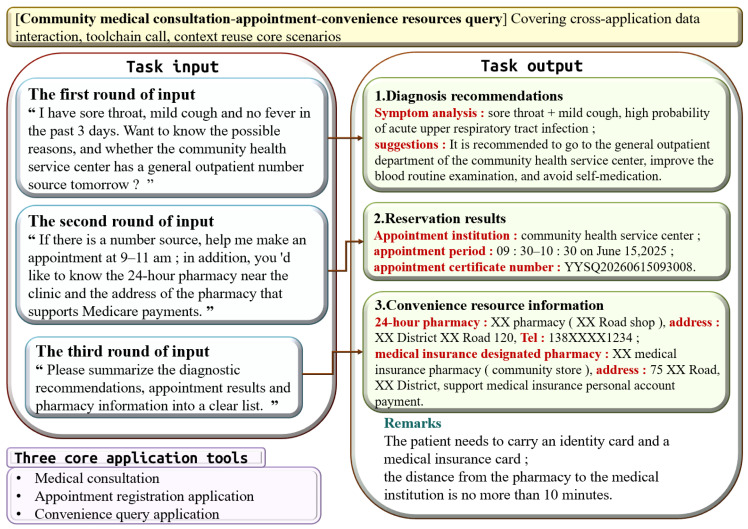
The input and output process diagram of multi-round collaborative task of community medical convenience service.

The multi-application collaborative community healthcare service scheduling task is designed to simulate the process in which users accomplish multi-turn collaborative tasks through a LLM agent. It encompasses the following three core functional modules: community medical consultation, appointment registration, and convenient resource search, which correspond, respectively, to the medical consultation, clinic appointment, and convenience search tools. The task consists of three turns of input as follows: the first turn queries symptoms and available appointment time slots, the second turn executes the appointment operation and searches for nearby pharmacy information, and the final turn summarizes the information. The specific inputs and outputs of the task are illustrated in Figure 5.

By performing a unified task instance under controlled variable conditions, it is possible to precisely compare operational performance across different environmental configurations. This task adopts a symbolic multi-application collaboration paradigm to simulate the collaborative operational scenarios of LLMs in real-world applications.

### 6.3. Quantitative Index Design

Four core quantitative metrics were selected to evaluate functional integrity, operational stability, and resource adaptability.

- **Task Completion Rate**: Determines whether the framework fully outputs all required task information.
- **Average Response Time**: Reflects the operational efficiency of the framework, measured in seconds (s).
- **Peak Resource Utilization**: Includes peak CPU utilization (%) and peak memory utilization (%), indicating how reasonably the framework utilizes limited resources.
- **Error Occurrence Rate**: Tracks the types of errors encountered during task execution, reflecting operational stability.

### 6.4. Experimental Procedure

First, in the experimental preparation phase, we ensure the host environment and the VMware virtualization environment are ready in terms of hardware, software, network, and storage. A dataset containing information such as community health center appointment slots and nearby pharmacy details is constructed and stored in Redis. Concurrently, parameters for the LLM-Conductor framework are configured: the Policy Memory Graph, dynamic resource allocation, and elastic toolchain scheduling are enabled, with *max_tokens* set to 512 and the number of PPO reinforcement learning iterations set to 100.

Next, we proceed with the deployment of gradient environments. Six virtual machines are created using VMware, with CPU, memory, GPU, and storage resources allocated according to the gradient specifications. A consistent system image, framework dependencies, and network synchronization are configured across all environments to ensure experimental consistency.

Following this, framework deployment and initialization are completed. The LLM-Conductor framework is deployed in all six gradient environments and linked to tools including Medical-Consult, Clinic-Booking, and Convenience-Query. Redis is initialized and loaded with the dataset. The monitoring-feedback closed-loop module is activated to verify successful framework initialization and normal operation.

Subsequently, task execution and data collection are carried out. Multi-application collaborative tasks are executed in each gradient environment based on three rounds of user input, repeated three times to mitigate random errors. Four types of quantitative metrics are collected synchronously as follows: task completion status and response times are recorded via framework logs; peak CPU and memory utilization are captured via the VMware monitoring center; and error types and frequencies are tallied from framework error logs.

Upon completion of data collection, the average values of the metrics from the three repeated trials are calculated to reduce variance. The differences in metrics across the six gradient environments are compared to analyze the impact patterns of computational resources on the framework’s functionality and performance.

Finally, based on the analysis results, conclusions are drawn as follows: the framework’s adaptation capability across different resource-constrained environments is determined, and its adaptation boundaries and applicable scenarios under such constraints are clarified.

### 6.5. Experimental Results and Analysis

**Figure 6:**
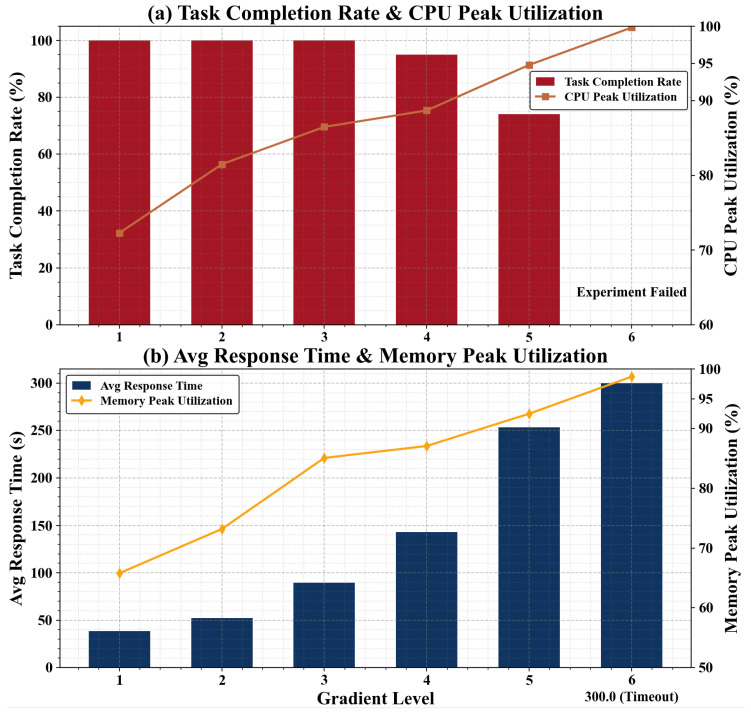
The trend chart of average response time, resource peak utilization and error rate under different gradient levels.

The experimental results are shown in Table 11 and Figure 6. The analysis is as follows:

**Gradient Variation in Task Completion Rate.** As the key indicator of core functionality adaptation, the task completion rate remains at 100% within the Gradient 1–3 range. During this phase, CPU and memory resources are sufficient to fully support the loading of the framework’s core modules and multi-application collaboration processes, with no loss of functional integrity. The rate decreases to 95% at Gradient 4, where only non-core formatting functions are missing while core tasks remain operational. A sharp decline to 74% occurs at Gradient 5, where the 256 MB memory limit leads to incomplete loading of the symptom diagnosis model, and the CPU frequency of 0.3 GHz causes timeouts in appointment logic calculations, resulting in core function errors. At Gradient 6, framework initialization fails due to the 128 MB memory constraint, and the inference engine cannot start with a CPU frequency of 0.2 GHz, causing the task completion rate to drop to 0%.

**Correlation Between Metrics and Computational Resources.** The average response time shows a linear increasing trend as computational resources diminish, rising from 38.6 s at Gradient 1 to 253.6 s at Gradient 5. The primary source of delay shifts from toolchain interactions to slowed computation and data loading caused by insufficient resources. By Gradient 6, tasks time out completely. Peak CPU and memory utilization consistently increase as resources are reduced. The Gradient 1–3 range maintains resource redundancy, Gradients 4–5 approach saturation, and Gradient 6 exhausts resources completely, triggering the forced termination of system processes.

**Adaptation Interval Classification.** The error occurrence rate shows a sharp negative correlation with computational resources. No errors occur in Gradients 1–2, while only minor errors in non-core functions appear in Gradients 3–4. The error rate rises to 40% at Gradient 5, involving core functionality, and reaches 100% at Gradient 6. Based on these data, the adaptation capability of LLM-Conductor in resource-constrained environments can be classified into the following four consecutive intervals: Stable Adaptation, Critical Adaptation, Core Function Errors, and Complete Failure, corresponding to Gradients 1–3, Gradient 4, Gradient 5, and Gradient 6, respectively. The defining characteristics of each interval are determined by both functional integrity and resource status.

**Adaptation Boundaries and Sensitivity.** Further analysis reveals that the core bottlenecks for adaptation in this experiment are memory and CPU frequency. Core functionality remains available when memory is greater than or equal to 512 MB and CPU frequency is greater than or equal to 0.5 GHz. Core function errors occur when memory is less than or equal to 256 MB and CPU frequency is less than or equal to 0.3 GHz. The framework becomes inoperable when memory is less than or equal to 128 MB and CPU frequency is less than or equal to 0.2 GHz. The sensitivity of metrics to changes in computational resources, from highest to lowest, is as follows: Task Completion Rate and Error Occurrence Rate (criteria for core functionality), Average Response Time (indicator of efficiency), and CPU/Memory Utilization (early warning indicators for resource bottlenecks).

### 6.6. Experimental Conclusions

In resource-constrained environments simulated via graded hardware reduction, the LLM-Conductor framework demonstrates excellent functional integrity, with its core functionality remaining largely unaffected. This indicates its suitability for deployment in most scenarios with limited resources. Furthermore, the framework exhibits strong stability under low-resource conditions: even in extreme configurations, no critical failures occur, and resource utilization remains within safe boundaries without causing system crashes or resource overflow.

In conclusion, we can reasonably predict that the LLM-Conductor framework exhibits robust adaptability in resource-constrained environments, with promising prospects for deploying multi-application collaborative tasks in edge computing, the Internet of Things (IoT), and other analogous resource-limited scenarios. These findings provide experimental support from simulated settings for its potential industrial-scale deployment.

## 7. Discussion

Ablation experiments isolate the marginal contributions of monitoring-decision coupling, structured memory, and joint resource optimization, indicating that gains stem from the proposed mechanisms rather than model capabilities. Horizontal comparisons against passive isolation, incremental decision-making, and fixed-quota baselines further demonstrate the differentiated competitiveness of active closed-loop monitoring, global planning, and dynamic co-optimization in industrial settings.

Graded resource-reduction tests identify the continuous operational boundaries from stable execution to functional failure under extreme constraints. Together, these three dimensions form a cohesive evidence chain, showing that LLM-Conductor unifies security, functionality, and resource efficiency by deeply coupling the traditionally isolated decision, memory, and resource layers, thereby offering an empirically supported pathway for reliable LLM deployment in IIoT environments.

Beyond the seven risk categories covered by the test suite, several adversarial blind spots remain as follows:

- **Indirect prompt injection via tool return contamination.** Current tests focus on direct user-input injection, yet attackers may embed malicious instructions within tampered search results or database returns, exploiting the monitoring-feedback loop to amplify attacks across turns.
- **Adversarial evasion of anomaly detection.** The suite lacks adversarial samples targeting Isolation Forest and DBSCAN; attackers could bypass detection via low-and-slow request patterns or log payloads statistically similar to benign traffic.
- **Policy memory graph poisoning.** Long-term adversarial interactions may inject false tool-parameter associations into RedisGraph, causing subsequent cross-task transfers to inherit poisoned policy topologies.
- **Monitoring side-channel leakage.** Zero-intrusion collection avoids host modification, yet attackers may infer security thresholds and toolchain configurations from response latency and scheduling patterns exposed by the feedback loop.
- **Compositional privilege escalation.** Cross-application permission tests do not cover sequential multi-tool exploits where individually benign tools, orchestrated in a crafted order, achieve system-level control.
- **Safety contraction under elastic degradation.** Resource-constrained degradation retains only lightweight tools, but the security boundary of this reduced toolset—without surrounding monitoring modules—has not been formally verified.

## 8. Conclusions

The core contribution of the LLM-Conductor architecture lies in providing a systematic and industrially viable technical pathway for LLM deployment. It breaks from the traditional paradigm of isolated optimization by deeply coupling decision-making policies with resource scheduling, thereby resolving the fundamental tension between the high computational demands of LLMs and the constraints of industrial environments. Implemented using LangChain and vLLM for open-source deployment, the framework supports both Command-Line Interface (CLI) and Application Programming Interface (API) invocation modes, ensuring compatibility with existing systems. Its *non-intrusive design* and capability for *resource adaptation* enable seamless deployment across cloud servers, edge nodes, and lightweight IoT devices, significantly expanding the application boundaries of LLM-powered agents.

**Limitations:** First, the scenario coverage is currently concentrated in consumer-grade domains (healthcare, transportation, and email), validated through software-layer interaction patterns rather than physical IIoT deployments. Second, the experimental environment relies entirely on VMware virtualized instances with emulated resource constraints; no real-world industrial sensors, actuators, or OT communication protocols (e.g., Modbus, Profinet) were integrated. Third, the convergence speed of reinforcement learning in high-dimensional dynamic environments remains relatively slow, and compatibility with multi-model heterogeneous deployment requires further investigation. These limitations delineate the current work as a software-layer architectural validation; physical-layer field tests on actual industrial testbeds are planned as immediate future work.

**Key solution approach:** To upgrade the current TF-IDF text-feature-based single matching mode to a hierarchical memory retrieval system combining semantic embedding, topological structure, and meta-policy separation, thereby enabling the memory graph with cross-domain abstraction and structural generalization capabilities. Specific improvements can be made along the following four complementary dimensions, all compatible with the existing architecture:

1. **Semantic encoding layer replacement.** Replace TF-IDF sparse vectors with lightweight Sentence-BERT dense embeddings to capture cross-domain intent similarity, using offline pre-computation and runtime cosine retrieval to maintain low latency in resource-constrained environments.
2. **Toolchain topological structure matching.** Augment task nodes with execution path signatures encoding historical tool invocation topologies, enabling policy reuse based on structural similarity rather than lexical matching alone.
3. **Meta-policy and domain parameter separation.** Introduce meta-policy nodes to store domain-agnostic decision patterns separately from domain-specific task parameters, enabling rapid cross-domain adaptation by reusing general policy skeletons.
4. **Adaptive similarity threshold.** Replace the fixed similarity threshold with a resource-aware dynamic mechanism that balances exploration and reuse according to real-time system load, integrating directly with the existing elastic scheduling module.

**Future Directions:** At the framework optimization level, we plan to construct a large-scale, cross-domain annotated dataset to expand the feature dimensions of the policy memory graph and refine the constraint modeling within the Markov Decision Process (MDP) state space, thereby improving adaptability to complex scenarios [40,41,42]. Meta-reinforcement learning algorithms will be introduced to optimize the policy training process, leveraging cross-task experience transfer to accelerate convergence in high-dimensional dynamic environments and reduce dependence on samples for online fine-tuning [41]. We will integrate model quantization, knowledge distillation, and lightweight toolchain pruning techniques to design adaptation schemes for resource-constrained embedded devices, balancing functional integrity with resource consumption [43,44]. Furthermore, we will extend a unified multi-model adaptation interface, establish a standardized monitoring metric system and resource scheduling policy for heterogeneous models, aiming to achieve autonomous optimization in multi-model collaborative deployment scenarios [45,46].

In addition, the LLM-Conductor architecture can be extended to industrial scenarios involving complex physical processes, such as environmental science. Recent research has proposed Hydro-Agent, a physics-integrated LLM multi-agent framework that has been successfully applied to autonomous inverse modeling of groundwater systems [47], demonstrating the significant potential of LLM multi-agents in scientific computing. Hydrogeological monitoring networks rely on field edge nodes and sensors, which naturally align with the resource-constrained and zero-intrusion deployment assumptions of this architecture. Future work will explore migrating the policy memory graph and elastic resource scheduling mechanisms to environmental sensing scenarios, supporting tasks such as hydrological parameter inversion and pollutant diffusion simulation through cross-domain meta-policy nodes, while leveraging the zero-intrusion monitoring-feedback closed loop to ensure stable access of field devices, thereby further validating the universality of the architecture in cross-domain Industrial Internet of Things environments.

## Figures and Tables

**Table 1:** Composition of the real-world scenario case set.

Task Name	Type	Tools Involved	Data Source
Travel Expense Calculation	Single-app	metro_hail, quick_ride	Simulated API
Email Summarization	Single-app	get_email	Real API
Medical-Tourism	Multi-app	health, travel_mate	Simulated backend
Symptom Consultation	Single-app	health_companion	Simulated API

Note: Travel Expense Calculation: Basic tool invocation and parameter accuracy; Email Summarization: Long-text comprehension and cross-session memory reuse; Medical-Tourism Collaboration: Cross-application data transfer and context consistency; Symptom Consultation: Knowledge-based Q&A and sensitive parameter handling.

**Table 2:** Comparison of two types of LLM systems, industry standards and optimization effects of core security assessment indicators under large-scale benchmark testing.

Metric	Vanilla-LLM-App	LLM-Conductor	Standard	Effect
ARIR	70.6%	1.3%	N/A	97.8%
AAET	826.4	Not Triggered	N/A	Best Effect
ADLV	3.2	0.02	N/A	99.4%
ADR	N/A	96.7%	≥90%	7.4%
DRT	N/A	78.9	≤100	21.1%
FPR	N/A	1.8%	≤5%	64.0%

Note: ARIR is the average incidence of risk; AAET is the average attack effective time, unit ms; ADLV is the average amount of data leakage, which refers to the average number of data leakage items in a single data leakage case; ADR is abnormal detection rate; DRT is the abbreviation of defense response time, unit ms; FPR is the false alarm rate. In the “Effect” column, ARIR and ADLV denote the reduction rates relative to Vanilla-LLM-App, calculated as (Vanilla value − Conductor value)/Vanilla value × 100%; ADR denotes the improvement rate relative to the industry standard, calculated as (Conductor value − Standard value)/Standard value × 100%; DRT and FPR denote the optimization rates relative to the industry standard, calculated as (Standard value − Conductor value)/Standard value × 100%; and AAET is marked as “Best Effect” because no attack was triggered in LLM-Conductor.

**Table 3:** Risk incidence rate of two LLM systems.

Risk Type	Number of Tests	Risk Incidence Rate (Wilson Score Range at 95% Confidence Level)	
		Vanilla-LLM-App	LLM-Conductor
MTT	320	72.0% (66.8–76.6%)	0.0% (0.0–1.2%)
SDE	320	69.3% (64.0–74.1%)	0.2% (0.03–1.56%)
UDL	300	65.2% (59.6–70.4%)	0.8% (0.24–2.60%)
FA-NLA	300	58.4% (52.8–63.8%)	2.3% (1.11–4.69%)
REA	280	81.5% (76.5–85.6%)	1.1% (0.38–3.15%)
IIA	242	76.8% (71.1–81.7%)	2.7% (0.38–3.15%)
CPA	242	73.6% (67.7–78.7%)	2.4% (1.09–5.20%)
Summary Analysis	Total	Average	
	2004	70.6% (68.65–72.59%)	1.3% (0.8%–2.0%)

Note: The abbreviation of risk type corresponds to the full name, MTT (malicious toolchain tampering), SDE (sensitive data theft), UDL (unintentional data leakage), FA-NLA (natural language ambiguity function abnormality), REA (resource exhaustion attack), IIA (instruction injection attack), and CPA (cross-application permission abuse); the value in the brackets after each risk rate is the Wilson score interval at the 95% confidence level, indicating that the real risk rate has a 95% probability of falling within this interval.

**Table 4:** Task completion performance comparison between Vanilla-LLM-App and LLM-Conductor across different task types.

Task Type	Vanilla-LLM-App		LLM-Conductor	
	Steps	Overall	Steps	Overall
SA-TCC	1.00	1.00	1.00	1.00
SA-ES	0.80	0.80	1.00	1.00
MAC-MT	0.29	0.40	1.00	1.00
SA-SC	0.90	0.90	1.00	1.00
LC-AI	0.95	0.95	0.89	0.97
LC-MAC	0.76	0.95	0.95	0.95

Note: SA-TCC corresponding to single application (traffic cost calculation); SA-ES corresponding to the single application (mail summary); MAC-MT corresponds to multi-application collaboration (medical-travel); SA-SC corresponding to single application (symptom consultation); LC-AI corresponds to LangChain without application dependence; and LC-MAC corresponds to LangChain multi-application collaboration.

**Table 5:** The error type distribution of the two types of architectures.

Error Type	Error Proportion	
	Vanilla-LLM-App	LLM-Conductor
ARI	14.3%	7.6%
EANI	14.3%	10.6%
OFE	28.6%	4.8%
CLE	2.4%	2.0%
CADM	28.6%	0.0%

Note: For clarity, the abbreviations of error types are expanded as follows: ARI corresponds to Application Repeated Invocation, EANI denotes Expected Application Not Invoked, OFE refers to Output Format Error, CLE stands for Context Length Exceeded, and CADM represents Cross-Application Data Mismatch.

**Table 6:** Comparison of response time and token resource utilization between Vanilla-LLM-App and LLM-Conductor under different task scenarios (mean ± standard deviation).

Task Type	Number	Vanilla-LLM-App		LLM-Conductor	
		Average Time (s)	Token Utilization	Average Time (s)	Token Utilization
NAQ	42	19.5 ± 2.3	35.2% ± 4.2	21.4 ± 2.1	85.2% ± 3.1
SAQ	20	32.0 ± 3.1	40.0% ± 3.8	39.2 ± 3.5	90.4% ± 2.7
MAQ	10	22.3 ± 2.8	30.3% ± 4.5	41.6 ± 4.2	88.9% ± 3.3
MACQ	21	31.7 ± 3.5	25.9% ± 5.1	59.9 ± 5.1	92.4% ± 2.5

Note: For clarity, NAQ means no application query; SAQ represents single application query; MAQ represents multi-application query; and MACQ represents multi-application queries.

**Table 7:** Security performance comparison (2004-case test set).

Metric	ISOLATEGPT	LLM-Conductor
Risk Incidence Rate	8.5%	1.3%
Anomaly Detection Rate	N/A (module absent)	96.7%
Defense Response Time	>2000 ms	78.9 ms
False Positive Rate	N/A	1.8%
Deployment Invasiveness	High	Zero-intrusion
Multi-App Collaboration Latency Overhead	+92.4%	<3%

Note: Risk Incidence Rate = (Triggered risks/2004 cases) × 100%. Anomaly Detection Rate = (Detected anomalies/Actual anomalies) × 100%, N/A for ISOLATEGPT. Defense Response Time = Mean ms from detection to execution; ISOLATEGPT exceeds 2000 ms (manual confirmation) vs. 78.9 ms automated in LLM-Conductor. False Positive Rate = (False alarms/Benign requests) × 100%, measured via parallel benign injection. Deployment Invasiveness: “High” for ISOLATEGPT (seccomp/setrlimit host modifications); “Zero-intrusion” for LLM-Conductor (strace: no write/ptrace). Multi-App Overhead = (Multi-app latency − Baseline)/Baseline × 100%; +92.4% for ISOLATEGPT (Hub-Spoke IPC + cold-start), <3% for LLM-Conductor.

**Table 8:** Functional integrity comparison.

Task Type	Step Accuracy		Completion Rate		CADM (%)		OFE (%)	
	LLM-C	ReAct	LLM-C	ReAct	LLM-C	ReAct	LLM-C	ReAct
SA-TCC	1.00	0.94	1.00	0.94	0.0	0.0	0.0	6.0
SA-ES	1.00	0.82	1.00	0.82	0.0	0.0	0.0	12.0
MAC-MT	1.00	0.42	1.00	0.52	0.0	22.0	0.0	18.0
SA-SC	1.00	0.86	1.00	0.86	0.0	0.0	0.0	8.0
LC-AI	0.96	0.90	0.98	0.90	0.0	0.0	2.0	6.0
LC-MAC	0.98	0.62	1.00	0.78	0.0	16.0	2.0	14.0

Note: Step Accuracy = (Correct tool invocations and parameter fillings/Total steps). Completion Rate = (Successful cases producing expected outputs/Total test cases). CADM (Cross-Application Data Mismatch Rate) = (Cases with data field errors or losses during cross-tool transmission/Total cross-application collaborative cases) × 100%. OFE (Output Format Error Rate) = (Cases with final outputs deviating from the expected Schema/Total cases) × 100%. All metrics are evaluated on the same dataset (LangChain Benchmarks and custom scenarios); step correctness is automatically determined by the LangChain Evaluator, while cross-application data consistency is manually verified.

**Table 9:** Comparison of Token Utilization Efficiency Across LLM Deployment Strategies.

Metric	CostBench	Vanilla-LLM	LLM-Conductor
Average Token Utilization	∼55%	32.9%	88.9%
MACQ Token Utilization	∼48%	25.9%	92.4%

Note: CostBench reports ab approximate token utilization under fixed-quota strategies for multi-turn tasks. Vanilla-LLM-App represents the fixed-quota measurement in the same environment. LLM-Conductor employs context-aware dynamic joint optimization. MACQ denotes Multi-Application Collaborative Queries.

**Table 10:** Hardware configurations for different resource-constrained gradients.

Gradient	CPU Config	Memory	GPU Config	Storage Config
1	4-core (4-thread) @2.0 GHz	8 GB	1 virtual GPU (2 GB)	128 GB SSD
2	2-core (2-thread) @1.5 GHz	4 GB	1 virtual GPU (1 GB)	64 GB SSD
3	1-core (2-thread) @1.0 GHz	2 GB	CPU-only computing	32 GB SSD (eMMC-sim)
4	1-core (1-thread) @0.5 GHz	512 MB	CPU-only computing	8 GB SSD (eMMC-sim)
5	1-core (1-thread) @0.3 GHz	256 MB	CPU-only computing	8 GB SSD (eMMC-sim)
6	1-core (1-thread) @0.2 GHz	128 MB	CPU-only computing	8 GB SSD (eMMC-sim)

Note: Gradient 1 simulates edge computing nodes, Gradient 2 simulates industrial edge terminals, Gradient 3 simulates intelligent IoT gateways, Gradient 4 simulates lightweight IoT devices, Gradient 5 simulates ultra-lightweight IoT terminals, and Gradient 6 simulates minimal IoT sensors. CPU configuration is the number of cores, threads, frequency limit. GPU configuration is the number of blocks, memory, where the gradient 3–6 only rely on CPU. The storage configuration is the capacity of the SSD, where the gradient 3–6 performs a speed limit simulation eMMC.

**Table 11:** The statistical results of performance metrics under different gradient levels (three repeated experiments).

Gradient	Completion	Response	CPU	Memory	Error
1	100.0%	38.6 s	72.3%	65.8%	0%
2	100.0%	52.1 s	81.5%	73.2%	0%
3	100.0%	89.4 s	86.5%	85.1%	3.3%
4	95.0%	142.8 s	88.7%	87.1%	6.7%
5	74.0%	253.6 s	94.8%	92.5%	40.0%
6	0.0%	-	99.8%	98.7%	100.0%

Note: Error details: Gradient 3, 1 tool call timeout (Convenience-Query delay); Gradient 4, 2 errors (1 tool call timeout; 1 incomplete output, missing resource contact); Gradient 5, errors including wrong diagnosis recommendation, failed appointment (source query timeout), missing resource address; and Gradient 6, process terminated (memory overflow + insufficient CPU after initialization, no valid output).

## Data Availability

The standardized benchmark datasets used in this study are publicly available: LangChain Benchmarks are accessible at https://langchain-ai.github.io/langchain-benchmarks (accessed on 20 January 2026), ISOLATEGPT-Sec is available at https://github.com/llm-platform-security/SecGPT/tree/IsolateGPT-AE (accessed on 28 January 2026), and InjecAgent is referenced from its original publication. The real-world scenario case set (transportation cost calculation, email summarization, medical-travel collaboration, symptom consultation), the 406 independently extended security test cases, and the evaluation scripts are publicly available at https://github.com/white520-black/LLM-Conductor-main (accessed on 9 April 2026). The email summarization task involves real QQ mailbox data; for privacy considerations, these specific inputs have been anonymized and replaced with synthetic equivalent data, while the tool invocation logic and evaluation scripts remain fully open.
